# Assessing Deceased-Donor Kidneys Through Posttransplant Survival Prediction Algorithms

**DOI:** 10.1053/j.ajkd.2025.07.016

**Published:** 2025-10-22

**Authors:** Vishnu S. Potluri, Jeremy Rubin, Jarcy Zee, Sarah J. Ratcliffe, Michael O. Harhay, Peter L. Abt, Emily A. Vail, Chirag R. Parikh, Roy D. Bloom, Alessandro Gasparini, Michael Crowther, David S. Goldberg, Peter P. Reese

**Affiliations:** Renal-Electrolyte and Hypertension Division (VSP, RDB), Department of Surgery (PLA), and Department of Anesthesiology and Critical Care (EAV), Perelman School of Medicine; Department of Biostatistics, Epidemiology and Informatics (JR, JZ, MOH), University of Pennsylvania; and Children’s Hospital of Philadelphia (JZ), Philadelphia, PA; Department of Biostatistics, University of Virginia, Charlottesville, VA (SJR); Division of Nephrology, Department of Medicine, Johns Hopkins University, Baltimore, MD (CRP); Red Door Analytics, Stockholm, Sweden (AG, MC); Division of Digestive Health and Liver Diseases, Department of Medicine, University of Miami, Miami, FL (DSG); and Vanderbilt Center for Transplant Science, Vanderbilt University Medical Center, Nashville, TN (PPR).

## Abstract

**Rationale & Objective:**

The Kidney Donor Risk Index (KDRI) is widely used to rank the quality of deceased-donor kidneys and is integrated into the U.S. kidney allograft allocation system. However, the KDRI has modest predictive accuracy for allograft survival, and recent revisions to the KDRI, which removed donor race and hepatitis C virus status, also revealed model calibration problems. This study aimed to evaluate novel approaches for predicting posttransplant allograft survival.

**Study Design:**

Retrospective cohort study using Organ Procurement and Transplantation Network data from May 1, 2007, through December 31, 2021.

**Predictors:**

(1) Donor demographic and clinical variables (established predictors); (2) longitudinal laboratory data from the donor’s terminal hospitalization, such as serum creatinine (new predictors); and (3) recipient clinical variables (new predictors).

**Setting & Participants:**

75,867 adult kidney recipients at U.S. centers.

**Outcomes:**

The primary outcome was time to all-cause allograft failure over 3 years. A secondary outcome was delayed graft function, defined as dialysis in the first week after the transplant.

**Analytical Approach:**

We implemented and compared machine-learning statistical models versus traditional modeling approaches (ie, proportional hazards for the primary outcome and logistic regression of the secondary outcome) that incorporated various combinations of predictors. The performance metrics used to assess discrimination were the integrated (time-dependent) area under the curve (AUC) for allograft survival and the AUC for delayed graft function. To assess calibration, we calculated Brier scores and visually compared the predicted outcomes with the observed ones. Predictive performance was assessed in a 20% testing data split.

**Results:**

Neither machine-learning models nor the addition of longitudinal laboratory data from the donor hospitalization to traditional models improved discrimination. For the primary outcome, the final model (named the Kidney Allograft Survival Index) used a proportional hazards modeling approach. Adding recipient variables improved model discrimination (integrated AUC, 0.68) and achieved excellent calibration for the overall cohort and subgroups. The final model for delayed allograft function used logistic regression, included recipient variables, and had an AUC of 0.75 with acceptable calibration.

**Limitations:**

No external validation.

**Conclusions:**

Improving the discrimination and calibration of kidney allograft survival prediction models is achievable by including recipient characteristics. These enhanced models have potential to improve the system of kidney allocation.

Challenges in the assessment of the quality of donated kidneys generate major problems for the organ transplant system. In 2023, approximately 8,182 (25%) of all 32,553 kidneys from deceased donors were discarded in the United States.^1^ Some of these organs could have provided clinical benefit to the nearly 90,000 people waiting for a kidney transplant. The Advancing American Kidney Health Initiative, the Organ Procurement and Transplantation Network (OPTN), and advocacy organizations (eg, National Kidney Foundation) have emphasized the need for reducing organ discard.^2,3^ Improving the ability to predict outcomes for donated kidneys without the use of social constructs such as race is a priority for the kidney transplant community.^4–7^

To help transplant professionals select appropriate kidneys for their patients, the OPTN uses the Kidney Donor Risk Index (KDRI) as a scoring tool. The KDRI predicts the composite risk of graft failure, defined as a return to dialysis, repeat transplant, or death, but has modest predictive accuracy (C statistic, 0.61).^8^ Every deceased-donor kidney in the United States receives a KDRI score, which is used during kidney allocation. The KDRI includes cross-sectional data obtained at the time of donation, including donor age, height, weight, cause of death, history of hypertension or diabetes, creatinine, and donation after circulatory death. Earlier KDRI versions also included race and hepatitis C virus (HCV) status.^8,9^ The most recently released version of the KDRI eliminated the variables of donor race and HCV status, but the OPTN’s mandate was to make no other improvements to the formula. Further, the technical documentation accompanying the proposal revealed shortcomings with the calibration of the KDRI.^6,10^ The report affirmed that these calibration problems were also present with the original KDRI, although the original publication on the KDRI does not describe calibration. For example, in the scenario of kidneys from deceased donors aged <45 years, the KDRI will often predict survival rates 15%−25% higher or lower than observed survival rates. Notably, these calibration problems were apparent only through the inspection of graphs that showed observed versus expected survival rates.^6^ Inaccurate prediction can cause clinicians to accept kidneys based on flawed projections of posttransplant survival and undermine the informed consent process when discussing posttransplant expectations with patients.

Three potential approaches hold promise for better kidney allograft outcome prediction. Longitudinal laboratory measures of donor organ function (eg, serum creatinine and aminotransferase levels, pH) might capture organ health, acute injury, or physiological management of the donor better than single values measured at the time of procurement. The second is the use of novel analytical approaches to predict transplant outcomes using a transplant registry dataset augmented by these longitudinal data from the donor hospitalization. Prior studies have shown that flexible machine learning–based models might provide a small improvement in the prediction of deceased-donor kidney quality.^11–13^ The third approach is a conceptual shift away from the paradigm that the “quality” or “value” of the donor organ is derived exclusively from donor characteristics. A different concept would reflect that an organ’s value, when assessed by predicted future function, is a feature of donor and recipient characteristics and their biological compatibility.^14,15^ If implemented, this approach to assessing an organ’s value would also enable integration of a specific recipient’s characteristics when predicting posttransplant outcomes.

Therefore, we aimed to test all 3 approaches to improving allograft survival prediction using longitudinal laboratory data from the deceased donors’ terminal hospitalizations and kidney transplant recipient characteristics from the OPTN.

## Methods

### Overview and Cohort Generation

This study used data from the OPTN. The OPTN data system includes data on all donors, waitlisted candidates, and transplant recipients in the United States, submitted by the members of the OPTN. The Health Resources and Services Administration of the U.S. Department of Health and Human Services provides oversight of the activities of the OPTN contractor.

Using data from the OPTN (detailed in Item S1), we assembled a retrospective cohort of adult (age ≥18 y) recipients of deceased-donor kidneys in the United States. The dataset also included longitudinal clinical data from the donors’ terminal hospitalizations. The study was approved by the University of Pennsylvania Institutional Review Board (approval no. 850256) and is reported according to the Transparent Reporting of a multivariable prediction model for Individual Prognosis Or Diagnosis statement.^16^

**Figure 1.**
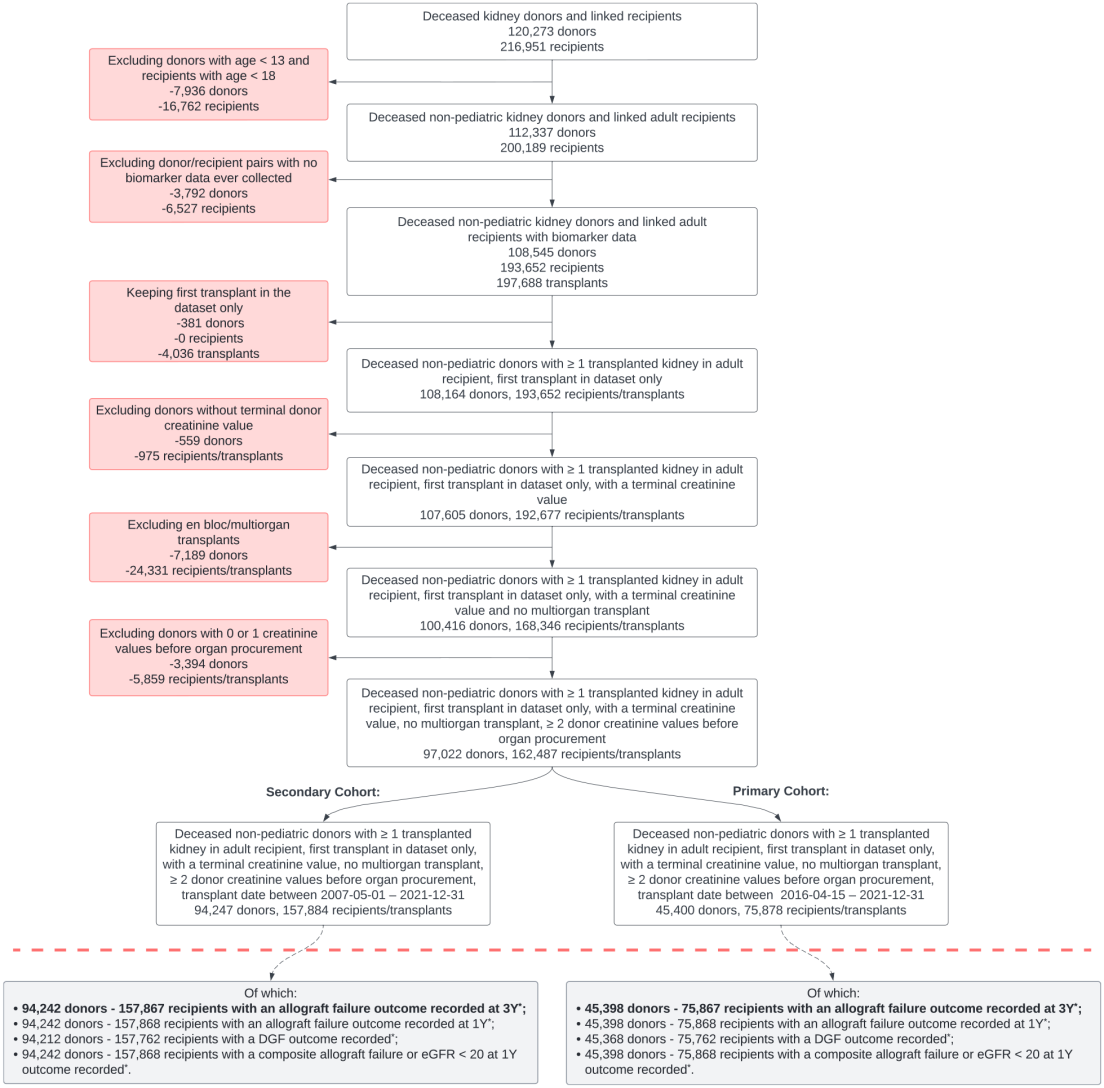
Flow chart depicting cohort generation. DGF, delayed graft function; eGFR, estimated glomerular filtration rate.

The primary cohort comprised adult kidney transplant recipients (age ≥18 y; Fig 1). We excluded transplants from donors aged <13 years because pediatric donors commonly have small stature or other organ-quality considerations related to physical immaturity. We further excluded donors without (1) collected longitudinal laboratory values, (2) terminal creatinine values, or (3) fewer than 2 creatinine values recorded during the terminal hospitalization (given the focus on longitudinal changes). If a patient received more than one kidney transplant during the study period, we analyzed only the first transplant episode. We excluded recipients of multi-organ transplants, dual kidney transplants, and en bloc kidney transplants. The primary cohort comprised transplants performed between April 15, 2016, and December 31, 2021. We selected the first date because April 2016 was when the OPTN mandated universal donor screening for active HCV infection with nucleic acid testing to detect HCV RNA, which is more sensitive and specific than the prior method of HCV antibody. In a secondary analysis, we assessed a limited set of models in a larger cohort of transplants performed between May 1, 2007, and December 31, 2021.

### Outcomes

The primary clinical study outcome was time to all-cause graft failure by 3 years after the transplant (ie, mortality, return to dialysis, or repeat kidney transplant). This outcome was used for development of the KDRI and is clinically meaningful to patients and transplant clinicians.^17^ Kidney recipients were censored on December 31, 2022 or the date of the last follow-up, whichever came first. The secondary study outcomes were delayed graft function (a binary outcome conventionally defined as receiving dialysis within the first week after transplant) and a combined binary outcome of 1-year graft failure or 1-year estimated glomerular filtration rate (eGFR) ≤20 mL/min/1.73 m^2^. The rationale for including eGFR <20 mL/min/1.73 m^2^ is that this level of kidney filtration function is low enough to qualify for the U.S. kidney transplant waiting list.^18^ Post hoc outcomes were: (1) 5-year all-cause allograft failure, (2) allograft failure by 3 years treating death as a competing risk using a Fine-Gray sub-distribution hazards model, and (3) a combined binary outcome of 3-year graft failure or 3-year eGFR ≤20 mL/min/1.73 m^2^ using a logistic regression model.

The race-neutral CKD-EPI (Chronic Kidney Disease Epidemiology Collaboration) equation was used to estimate recipient GFR,^19^ and it was calculated using the R statistical package “nephro.”

### Donor and Recipient Variables

We fit a series of models that comprised donor characteristics from the terminal hospitalization (“cross-sectional” variables available at the time of cross-clamp), donor longitudinal laboratory variables, allograft variables, and recipient variables. We selected the variables of serum creatinine, serum urea nitrogen, sodium, alanine and aspartate aminotransferases, International Normalized Ratio, total bilirubin, and albumin to test based on existing literature, clinical judgment, and experience with the strengths and limitations of registry data.^8,13,20–22^ We examined donor longitudinal laboratory values from the donor hospitalization collected within 48 hours before cross-clamp and last recorded (“terminal”) values and selected donor variables known or hypothesized to be associated with kidney allograft outcomes. The events leading to brain death and hospitalization (eg, trauma, stroke, hemodynamic instability) could lead to systemic ischemia, or brain death itself could cause systemic inflammation. Either mechanism can cause multiple-organ injury, affecting these laboratory measurements.^20,23–28^ We limited the data to a 48-hour window because of variations in the duration of the terminal hospitalization data; most donors had data recorded for ≥48 hours. Longitudinal laboratory data were transformed into summary data such as mean, median, highest, lowest, and slope values; these summary statistics were included in the analyses. Tables S1 and S2 list the variables tested and selected for the final models. In descriptive analyses, rates of missingness are reported and were not imputed.

Because the value of these analyses would be greater if the predictive algorithms could be made available to transplant clinicians for real-world use, we elected not to address missing data with multiple imputation. That approach is suitable for explanatory statistical models but poses challenges for user-friendly predictive modeling. Instead, we imputed missing data as means or medians, stratified by donor and recipient age quartiles, for binary and continuous covariates, respectively. Imputation values were defined using the primary cohort (described in Item S1).

### Development of Prediction Model and Internal Validation

The study cohort was divided randomly into training (80%) and testing (20%) data splits. We trained the models on the 80% training data split. For the primary outcome of 3-year graft failure, we compared a Cox regression model including only covariates in the revised KDRI with models with varying groups of predictors analyzed using Cox regression and 4 supervised machine-learning methods: random forest, LASSO (least absolute shrinkage and selection operator), ridge, and elastic net. Similarly, for the 2 secondary outcomes (delayed graft function and 1-year allograft failure or eGFR ≤20 mL/min/1.73 m^2^), we fit logistic models with the same covariate groups and compared them with the same 4 machine-learning approaches. For each approach, we fitted a series of models (designated models A-G) with different combinations of variables, as shown in Table 3 and Table S2. Model A is the basic model comprising the 8 variables in the revised KDRI.^10^ Model B added further cross-sectional donor variables to model A. Model C added longitudinal donor laboratory values to model B. Model D added allograft and recipient variables to model C. Model E added donor–recipient interactions (ie, statistical assessment for effect modification) to model D. Model F included 8 donor variables from the revised KDRI plus additional donor, recipient, and allograft variables. Model G added donor–recipient interactions to model F. Item S2 and Tables S1 and S2 provide additional details related to covariate group inclusion.

We performed 5-fold cross-validation using the entire training data to pick the regularization parameter for LASSO, ridge, and elastic net as well as node size for random forest, which optimized the C statistic. All models were then fit using the entire training data for their respective optimal tuning parameters. We then tested the final models on the 20% testing data split. Item S3 provides additional description of the machine-learning approaches.

### Performance Metrics

The primary performance metrics to assess discrimination were the integrated (time-dependent) area under the curve (iAUC) for allograft survival and AUC for logistic regression.^29^ To assess calibration, we calculated Brier scores and generated curves comparing predicted versus observed outcomes. Discrimination and calibration were reported from the 20% data split. Item S4 provides a more detailed discussion of measures to assess model performance.

All statistical analyses were performed in R (version 4.3; R Foundation for Statistical Computing).^30^ Machine learning methods were implemented using R packages “glmnet” (for LASSO, ridge, and elastic net models) and “randomForest.” Calibration curves were created using R packages “riskRegression” (for allograft survival outcomes) and “CalibrationCurves” (for delayed graft function).

## Results

Figure 1 shows how the cohorts were assembled. For the primary outcome of 3-year allograft failure, the primary cohort consisted of 75,867 deceased-donor kidney transplant recipients with 3-year outcome data. Tables 1 and 2 show the demographic and clinical characteristics of the donors, allografts, and recipients. The median recipient age was 54 (IQR, 44–64) years; 40% were female, 34% were of Black race, and 93% were receiving chronic dialysis at the time of transplant.

Table S3 shows that 30% of recipients experienced delayed graft function, and the rates of graft failure were 64 and 56 per 1,000 person-years at 1 year and 3 years after transplant, respectively.

Table 3 shows the performance metrics for the models of the primary outcome of 3-year allograft failure. These analyses demonstrate that the best iAUC was 0.68, and similar discrimination was achieved by Cox regression and the 4 machine-learning approaches. The addition of longitudinal donor laboratory data did not improve the predictive accuracy of the models. For example, the iAUC for the Cox analysis with model B (donor variables only) was 0.62 and did not change when longitudinal laboratory values were added in model C.

Notably, adding donor–recipient interactions (cytomegalovirus, human leukocyte antigen, age, sex, and cold ischemia time) did not improve discrimination (models E and G). The most important stepwise gains in discrimination resulted from adding multiple recipient variables (model D). The addition of age alone increased the iAUC only from 0.62 to 0.63. Our group selected model F (which included donor, allograft, and recipient variables that are feasible to record and confirm in real-world practice) as the final model and named it the Kidney Allograft Survival Index. This model had an iAUC of 0.678 (95% bootstrapped confidence interval, 0.677–0.679).

**Figure 2.**
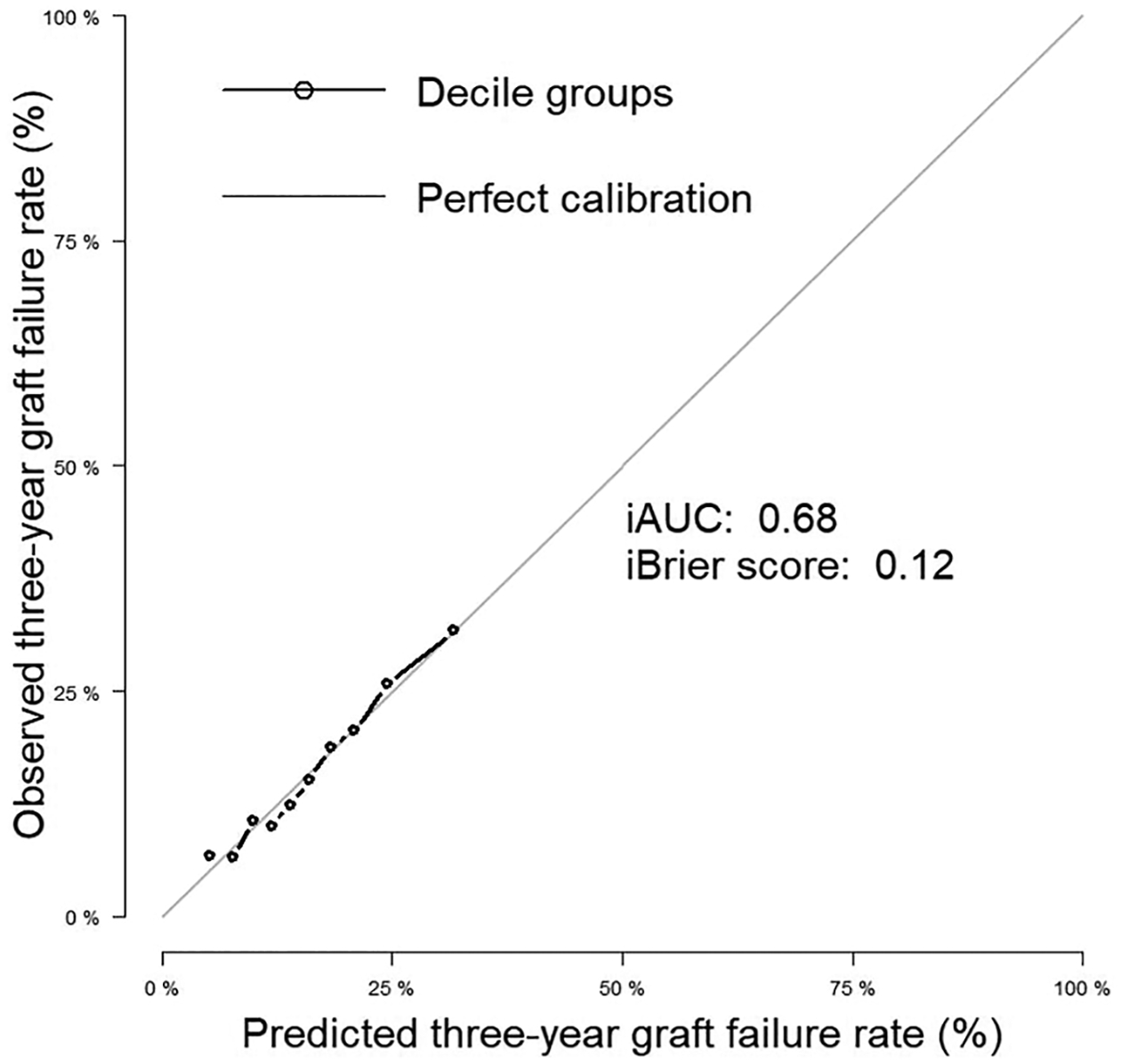
Calibration plot for the Cox regression model for 3-year allograft survival in the primary cohort (20% testing data split). Calibration assesses how close a risk score’s prediction of the expected outcome is to the observed outcome. iAUC, integrated (time-dependent) area under the curve.

**Figure 3.**
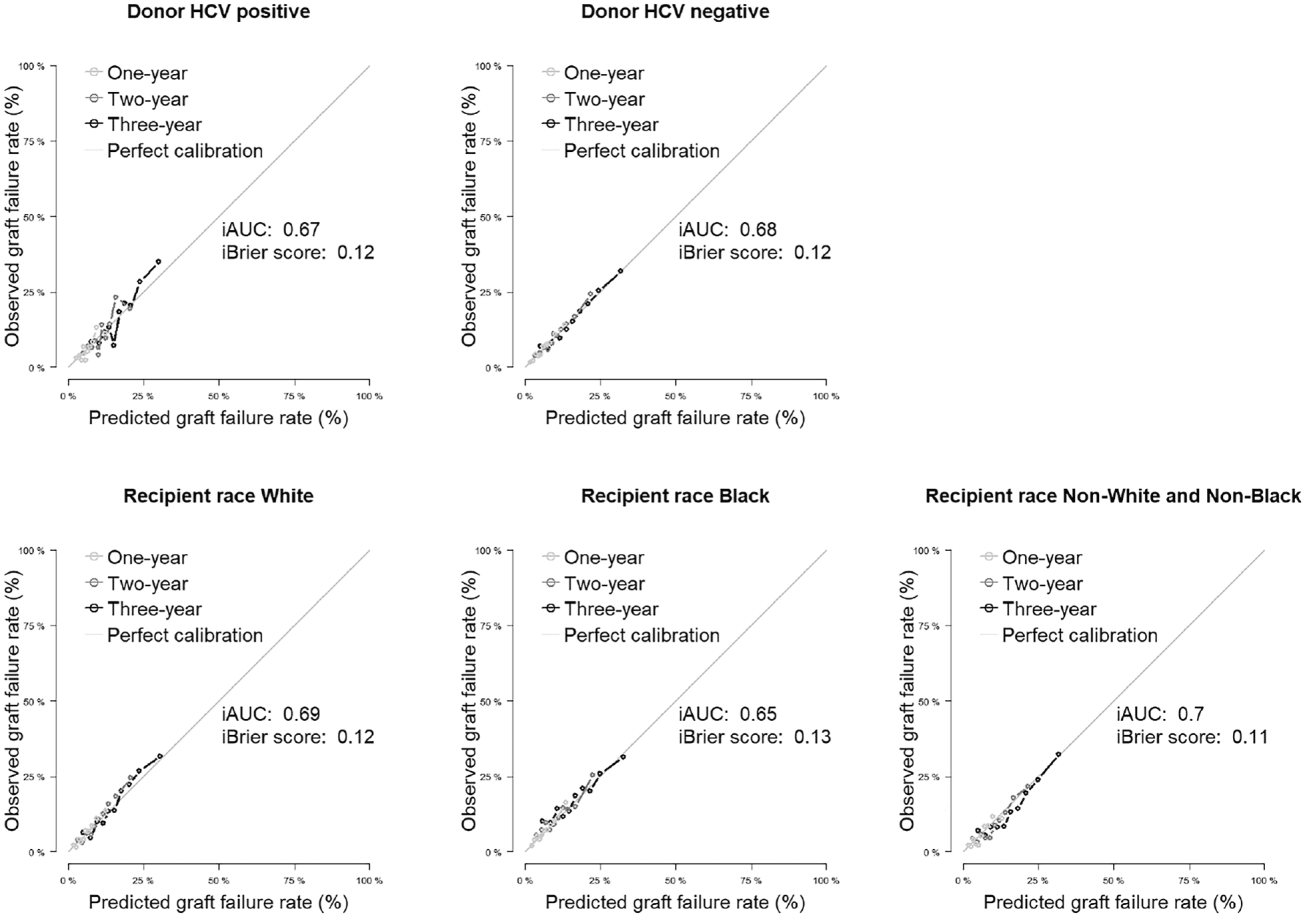
Calibration plots for 3-year allograft survival in selected subgroups based on donor hepatitis C virus infection and recipient race (20% testing data split). iAUC, integrated (time-dependent) area under the curve; HCV, hepatitis C virus.

**Figure 4.**
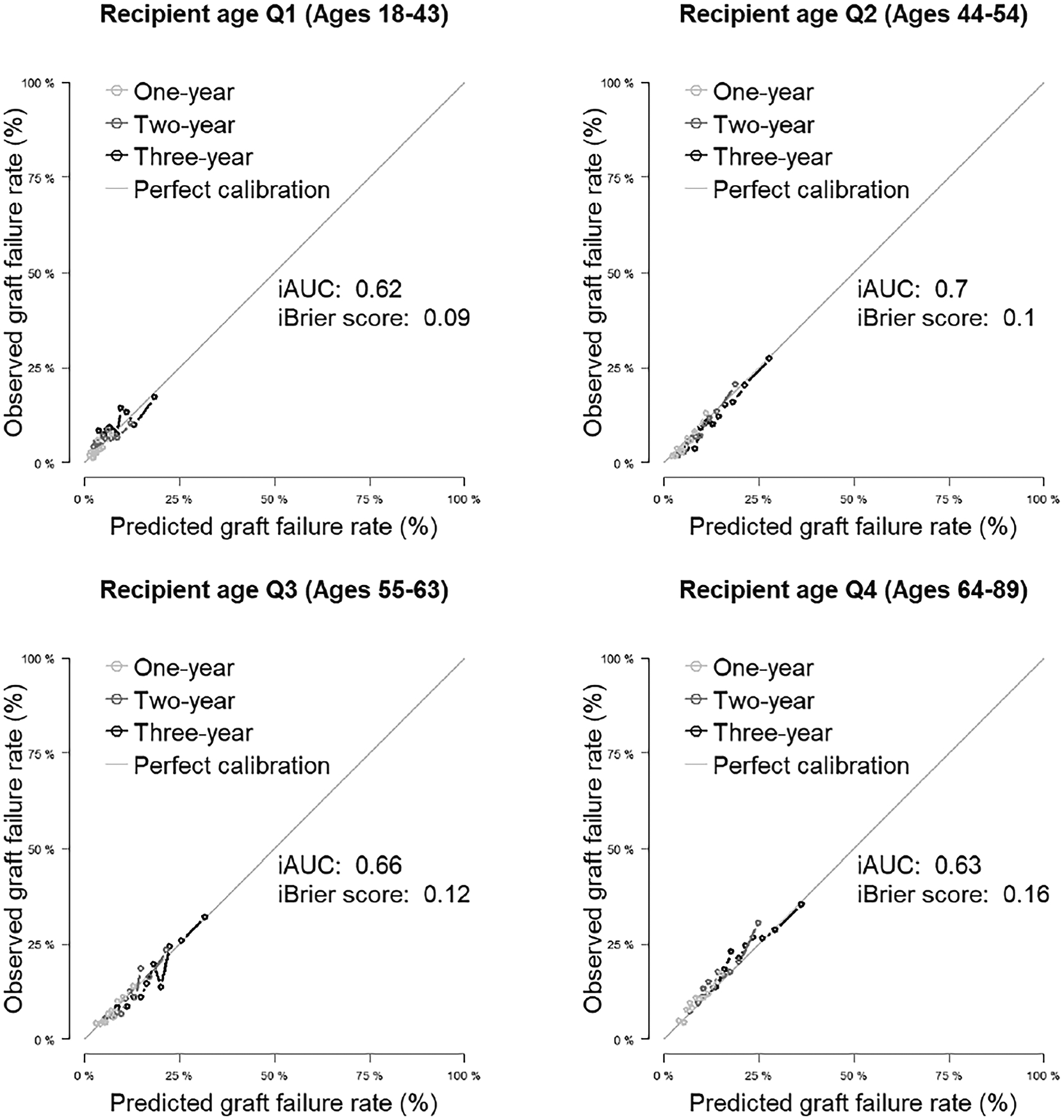
Calibration plots for 3-year allograft survival in selected subgroups based on recipient age (20% testing data split). iAUC, integrated (time-dependent) area under the curve.

All models consistently showed integrated Brier scores of ≤0.13. However, evaluating calibration requires additional evidence beyond Brier scores. For example, robust calibration requires close inspection that demonstrates that the predicted and observed rates of the outcome are similar overall and in clinically important subgroups.^31^ Figure 2 shows overall calibration (Fig S1 magnifies the calibration plot; Fig S2 shows a plot of calibration slope and intercept for the overall cohort), and Figs 3 and 4 display excellent calibration curves for model F among subgroups.

### Secondary Outcomes

Analyses of delayed graft function using the test data split demonstrated very good discrimination (Table S4). Adding recipient variables substantially improved the AUC, similar to the allograft survival models. Final AUCs were consistently 0.74–0.76. The incorporation of longitudinal donor laboratory values had minimal effect on predictive accuracy. The machine-learning approaches were not superior to logistic regression. The final logistic regression model using model F variables (donor and recipient variables but no longitudinal laboratory values or donor–recipient interactions) had an AUC of 0.75 and acceptable calibration with a Brier score of 0.18.

Prediction of the composite binary outcome of 1-year allograft failure or eGFR ≤20 mL/min/1.73 m^2^ using logistic regression generated discrimination at least as good as the machine-learning approaches. The AUC for this 1-year outcome using logistic regression was 0.68, with good calibration demonstrated by a Brier score of 0.07 using model F covariates (Table S5).

### Post Hoc Analyses

In a Cox regression analysis of the outcome of 5-year all-cause allograft failure, model F yielded an iAUC of 0.67 and an integrated Brier score of 0.18 (Fig S3). The Fine-Gray analysis of graft survival by 3 years with death as a competing risk using model F variables had an iAUC of 0.64 and a Brier score of 0.06. A logistic regression analysis of the composite binary outcome of 3-year allograft failure or eGFR ≤20 mL/min/1.73 m^2^ using model F variables yielded an AUC of 0.67 and a Brier score of 0.06.

### Secondary Cohort

In the secondary cohort that comprised transplants from the years 2007–2021, the iAUC for 3-year allograft survival using Cox regression and the same covariates (model F) was 0.65.

### Hypothetical Patient Examples

Table 4 provides examples of 3 hypothetical donors and 6 recipients to illustrate the effects of recipient and allograft characteristics on outcomes for the same donor using Cox regression and covariates from our final model (model F).

## Discussion

Accurate assessment of the suitability of deceased-donor organs for transplant is fundamental to efficient and equitable allocation, as well as informed consent for patients who must quickly decide whether to accept an offered organ or continue waiting. During the past 15 years, the U.S. transplant community has relied heavily on the KDRI for kidney quality assessment despite modest discrimination and calibration problems that were recently revealed.^6^ Our comprehensive analysis tested the value of machine-learning methods, longitudinal laboratory data from the donor’s terminal hospitalization, and the addition of recipient variables to improve the accuracy of allograft outcomes prediction. Although predictive accuracy was improved neither by incorporating novel data nor the use of machine-learning analysis, we demonstrated substantial improvement in predictive accuracy after including recipient variables. Implementing the Kidney Allograft Survival Index that comprises donor, allograft, and recipient variables into allocation rules and informed consent discussions would require a conceptual shift in how the transplant field estimates the quality of an organ. Still, we believe this endeavor is worthwhile and has been discussed in the past.^14,15,32^ This study brings novel data demonstrating that including recipient variables may also offer the most feasible way to achieve acceptable model calibration.

Many limitations of the KDRI have been described. In the United States, >90,000 individuals are on the kidney transplant waiting list, and many groups, such as individuals >60 years of age, are more likely to die than ever receive a transplant.^33^ In this setting, the high and increasing rate of discards at >25% of deceased-donor kidneys represents a waste of health care resources. The discards also disappoint the decedents’ next of kin.^1^ Despite limited predictive accuracy for allograft survival, the KDRI has a strong effect on the kidney discard rate, such that there is a dose–response relationship between worse KDRI scores and discard.^34,35^ The effect of poor KDRI scores as a “label” that may encourage discard was demonstrated in the year 2016 in a real-world setting, when a brief programming error caused miscalculation of KDRIs during organ allocation. Kidneys that crossed the threshold of “worst scoring 15% of kidneys” as a result of the error had an odds ratio for discard of 1.49 compared with when the KDRI was accurately calculated.^35^ It is possible that replacing the KDRI with a tool with better predictive accuracy such as the Kidney Allograft Survival Index might help clinicians to better identify appropriate kidneys that match their patients’ expectations for posttransplant survival. For example, even though the overall rate of organ discard might not change, the Kidney Allograft Survival Index could help clinicians more accurately identify those organs at highest risk for allograft failure for specific recipients. We acknowledge that kidney allocation is a complex and challenging process, and it is unknown whether adoption of this new Kidney Allograft Survival Index would affect the nonuse rate of deceased-donor kidneys.

Notably, the KDRI represents a conceptual framework that assumes that good kidney quality can be assessed as predicted survival using a statistical model. Alternative concepts include defining quality through histological samples, measuring urine or perfusate inflammatory biomarkers, or responding to a physiological challenge such as resistance during machine perfusion.^36,37^ Unfortunately, recent attempts to increase the accuracy of prediction of allograft survival by adding biopsy results to a model with donor characteristics did not demonstrate substantial improvements in discrimination.^38,39^

During the past few years, the OPTN and the Scientific Registry of Transplant Recipients (SRTR) collaborated to revise the KDRI.^6,10^ This revision was primarily motivated by a response to valid critiques that the negative valence of donor Black race created ethical concerns^40,41^ and the negative valence of donor HCV likely did not reflect the recent success of direct antiviral agents in curing HCV after transplant.^42,43^ The new 8-variable KDRI achieved similar discrimination as the earlier 10-variable KDRI, but the calibration plots in the SRTR technical reports reveal major limitations in accuracy.^10^ Calibration assesses for differences in the rates between the observed and predicted outcomes. In the SRTR report, one example of calibration problems is that the graph of 5-year survival for donors aged <45 years revealed multiple scenarios in which the predicted allograft failure risk differed from the observed risk by 10%−20%. This lack of calibration could undermine sound and well-informed transplant decision-making because kidneys with a model-predicted risk of failure of 30% at 5 years would have an observed failure rate of 55%.^6^ Taken together, these recent problems emphasize the need for new approaches to assessing organ quality.

In our analyses, we propose several explanations for the failure of donor terminal hospitalization data and machine-learning methods to improve prediction performance. For the former, it is possible that the donor terminal hospitalization data were collected over too brief a period or were confounded by the effects of treatment to reveal insights into donor quality. For example, trends in serum creatinine level might be expected to reveal long-term kidney injury in a donor. In our models, we tested minimum, maximum, and slope of creatinine level. However, laboratory measures of renal function might temporarily improve with fluid boluses or worsen with vasopressor medications. The quality of the data from the terminal hospitalization might also vary depending on the practices of organ procurement organizations or the willingness of hospital staff to measure or record laboratory values frequently. Selection bias could also emerge because only some kidneys are selected for transplant, likely including those with biomarkers with favorable profiles. Machine-learning approaches had similar discrimination as Cox and logistic regression models, although, in some cases, such as delayed graft function prediction, the random forest models demonstrated particularly poor calibration. These findings that machine-learning methods did not improve survival models in kidney transplantation are consistent with some prior publications, although these did not make use of longitudinal data from the donor’s terminal hospitalization.^13,44^ We acknowledge that machine-learning methods are also not well suited to all functional forms of longitudinal exposure data; it is possible that other methods such as joint models would achieve better predictive accuracy.

The main policy implication of these results is that the OPTN should explore the potential of integrating allograft prediction models with recipient variables into kidney allocation. This conceptual shift implies that the value that an organ provides to recipients is not determined only by donor attributes but instead must consider the recipient’s attributes and compatibility between the two. Aspects of donor and recipient compatibility might relate to their ages, human leukocyte antigens, sizes, or other characteristics. Using the analogy of a car buyer, a car’s value must be assessed in terms of expected performance for a specific driver profile. For example, a 2-door coupe may have little value to a driver who is 6 feet, 6 inches tall. In the future, transplant programs may be presented with organ offers in which the quality, as assessed by predicted outcomes, would vary according to recipient characteristics. This change in the format of organ offers might require a shift in informed consent requirements. For instance, instead of requiring patients to opt in for kidney offers for which the kidney donor profile index was >85%, the policy could be adapted to require opt-in for kidney offers for which the expected 3-year probability of graft failure is >25%.

Table 4 provides examples. For kidneys from donor 1 (small stature), differences in recipient height (tall vs below-average) had only minimal effects on predicted 3-year allograft survival accuracy. By contrast, kidneys from donors 2 and 3 show substantially higher 3-year predicted allograft failure rates when transplanted into older versus younger recipients. Donor 4 illustrates how allograft and recipient characteristics also strongly affect 3-year failure and delayed graft function rates.

One potential advantage of the Kidney Allograft Survival Index (vs traditional KDRI or the kidney donor profile index) is its ability to predict individualized 3-year allograft survival, improving clarity of communication between clinicians and patients by grounding decisions in a straightforward outcome. Although sharing predictive information with patients and transplant programs could improve informed consent, the optimal use and presentation of predictions derived from our study model would require collaboration between OPTN and SRTR researchers. Advice from patient groups, and perhaps integration into decision aids, might also be needed to test this approach’s value for stakeholders.^45^ Centers would also need to maintain up-to-date clinical information about their waitlisted patients for use in the model when fielding organ offers.

We acknowledge limitations of the present work. Although we used rigorous methods to test allograft prediction using training and test splits of OPTN registry data, the analyses could have benefited from external validation. OPTN registry data have known shortcomings, particularly regarding ascertaining recipient attributes and mortality outcomes.^46^ By contrast, we took advantage of recently updated datasets that have substantially improved mortality data, and by limiting outcomes to a 3-year time horizon, we think it is likely that nearly all deaths and allograft failures would have been reported to centers and captured in the data. We also acknowledge that discrimination in the range of 0.68 for 3-year graft survival leaves substantial room for improvement. Nonetheless, gains in discrimination and calibration over the existing KDRI could lead to meaningful benefits. Potentially promising future directions to improve discrimination could include more detailed phenotyping of donor comorbidities from the entire electronic medical record, noninvasive imaging of donor kidneys such as with quantitative ultrasonography, using artificial intelligence to better standardize biopsy light microscopy interpretation, or applying molecular methods such as transcriptomics to better phenotype tissue injury in the biopsy.

In summary, the accurate prediction of allograft survival after kidney transplant is central to the efficient allocation of deceased-donor kidneys and the processes of informed consent. The current KDRI has modest discrimination and poor calibration. The present study offers additional rigor compared with prior studies of kidney allograft prediction by providing insight into the calibration of the KDRI versus the proposed Kidney Allograft Survival Index. Kidney allograft prediction models that integrated donor, allograft, and recipient variables had substantially better discrimination than the revised KDRI and good calibration. This approach should be explored by transplant policymakers and professional societies committed to improving the national kidney transplant system.

## Supplementary Material

Supplementary Material **Figure S1:** Magnified calibration plot for the Cox regression model for the primary outcome of 3-year all-cause graft failure (the Kidney Allograft Survival Index) and the primary cohort (20% testing split).
**Figure S2:** Linear regression analysis of observed versus predicted 3-year all-cause graft failure (the Kidney Allograft Survival Index) and the primary cohort (20% testing split).
**Figure S3:** Calibration plot for the Cox regression model for the outcome of 5-year all-cause graft failure in the primary cohort (20% testing split); a post hoc analysis.
**Item S1:** Additional information about the dataset.
**Item S2:** Imputation methods used to handle missing donor and recipient data.
**Item S3:** Additional description of and rationale for machine-learning approaches.
**Item S4:** Further description of performance metrics of discrimination and calibration.
**Table S1:** List of donor and recipient variables tested.
**Table S2:** Variables in the final models.
**Table S3:** Cumulative incidence of outcomes among primary cohort of recipients with transplants recorded between April 15, 2016, and December 31, 2021, and secondary cohort of recipients with transplants recorded between May 1, 2007, and December 31, 2021.
**Table S4:** Discrimination and calibration for machine-learning and logistic regression analyses of the delayed graft function outcome.
**Table S5:** Discrimination and calibration for the secondary composite outcome of 1-year allograft failure and/or eGFR <20 mL/min/1.73 m^2^.

Supplementary File (PDF)

## Figures and Tables

**Table 1.** Characteristics of Deceased Kidney Donors by 3-Year Allograft Failure

		3-y Graft Failure	
Donor Characteristic	Overall (N = 75,867)^a^	Yes (n = 9,509)	No (n = 66,358)
Age, y	40 (29–52)	46 (34–55)	40 (29–51)
Female sex	28,566 (38%)	3,920 (41%)	24,646 (37%)
Height, cm	173 (165–180)	170 (165–178)	173 (165–180)
Weight, kg	82 (70–98)	82 (70–98)	82 (70–98)
Race/ethnicity			
White	51,872 (68%)	6,315 (66%)	45,557 (69%)
Black	9,909 (13%)	1,497 (16%)	8,412 (13%)
Hispanic	11,243 (15%)	1,349 (14%)	9,894 (15%)
Other/unknown	2,843 (3.7%)	348 (3.7%)	2,495 (3.8%)
Diabetes	6,094 (8.1%)	1,107 (12%)	4,987 (7.6%)
Hypertension	22,599 (30%)	3,646 (39%)	18,953 (29%)
History of smoking	15,924 (21%)	2,345 (25%)	13,579 (21%)
HCV RNA-positive	4,078 (5.4%)	490 (5.2%)	3,588 (5.4%)
HCV antibody positive	6,610 (8.7%)	800 (8.4%)	5,810 (8.8%)
Cause of death (cerebrovascular/stroke)	17,684 (23%)	2,763 (29%)	14,921 (22%)
Donation after circulatory death	20,277 (27%)	2,783 (29%)	17,494 (26%)
Terminal creatinine, mg/dL	0.97 (0.70–1.47)	1.00 (0.70–1.50)	0.96 (0.70–1.46)
Kidney donor profile index	0.43 (0.22–0.65)	0.54 (0.33–0.74)	0.42 (0.21–0.64)

Data presented as median (IQR) when applicable. Note that the unit is the kidney, so a single donor can contribute more than once to the data summarized in this table. Transplants were recorded between April 15, 2016, and December 31, 2021. Abbreviation: HCV, hepatitis C virus.

a Missing values: donor history of smoking (3,438; 2.27%), donor hypertension (1,744; 1.15%), donor diabetes (1,556; 1.03%), donor weight (164; 0.11%), donor HCV RNA positive (24; 0.02%), donor HCV antibody positive (18; 0.01%), and donor height (8; 0.01%).

**Table 2.** Kidney Transplant Recipient and Allograft Characteristics by 3-Year Allograft Failure

		3-y Graft Failure	
Characteristic	Overall (N = 75,867)^a^	Yes (n = 9,509)	No (n = 66,358)
**Recipient-level**			
Age, y	55 (44–64)	60 (50–67)	54 (43–63)
Female sex	29,980 (40%)	3,410 (36%)	26,570 (40%)
Height, cm	170 (163–178)	170 (163–178)	170 (163–178)
Weight, kg	82 (69–96)	84 (71–98)	81 (68–95)
**Race**			
White	27,582 (36%)	3,429 (36%)	24,153 (36%)
Black	25,871 (34%)	3,650 (38%)	22,221 (33%)
Hispanic	15,088 (20%)	1,672 (18%)	13,416 (20%)
Other/unknown	7,326 (9.7%)	758 (8.0%)	6,568 (9.9%)
**Cause of ESKD**			
Diabetes	22,775 (30%)	3,830 (40%)	18,945 (29%)
Hypertension	17,943 (24%)	2,138 (22%)	15,805 (24%)
Glomerular disease	10,043 (13%)	886 (9.3%)	9,157 (14%)
Cystic disease	5,629 (7.4%)	485 (5.1%)	5,144 (7.8%)
Other/missing	19,477 (26%)	2,170 (23%)	17,307 (26%)
Dialysis before transplant	69,293 (91%)	9,000 (95%)	60,293 (91%)
Dialysis duration, d	1,493 (716–2,323)	1,670 (931–2,472)	1,469 (690–2,299)
**Dialysis duration category**			
None	6,574 (8.7%)	509 (5.4%)	6,065 (9.1%)
≤3 y	21,385 (28%)	2,353 (25%)	19,032 (29%)
3–5 y	18,302 (24%)	2,409 (25%)	15,893 (24%)
>5 y	29,606 (39%)	4,238 (45%)	25,368 (38%)
Panel reactive antibodies	0 (0–48)	0 (0–43)	0 (0–48)
Prior transplant	8,619 (11%)	1,010 (11%)	7,609 (11%)
Diabetes	28,588 (38%)	4,719 (50%)	23,869 (36%)
History of cancer	7,170 (9.5%)	1,060 (11%)	6,110 (9.2%)
**Allograft-level**			
Cold ischemic time, h	18 (12–23)	18 (13–24)	18 (12–23)
**Donor sidedness**			
Left	36,277 (48%)	4,693 (49%)	31,584 (48%)
Right	39,590 (52%)	4,816 (51%)	34,774 (52%)
**HLA mismatches**			
0	3,736 (4.9%)	373 (3.9%)	3,363 (5.1%)
1	958 (1.3%)	123 (1.3%)	835 (1.3%)
2	3,716 (4.9%)	400 (4.2%)	3,316 (5.0%)
3	10,873 (14%)	1,290 (14%)	9,583 (14%)
4	21,110 (28%)	2,521 (27%)	18,589 (28%)
5	24,440 (32%)	3,237 (34%)	21,203 (32%)
6	11,034 (15%)	1,565 (16%)	9,469 (14%)
**Cytomegalovirus mismatch**			
Donor positive, recipient negative	13,730 (18%)	1,937 (20%)	11,793 (18%)
Donor positive/negative/unknown, recipient positive	51,422 (68%)	6,407 (67%)	45,015 (68%)
Donor negative, recipient negative	9,958 (13%)	1,077 (11%)	8,881 (13%)
Donor unknown, recipient negative/unknown	757 (1.0%)	88 (0.9%)	669 (1.0%)

Transplants were recorded between April 15, 2016, and December 31, 2021. Data presented as median (IQR) where applicable. Abbreviations: ESKD, end-stage kidney disease; HLA, human leukocyte antigen.

a Missing recipient-level values: recipient history of cancer (478; 0.32%), recipient diabetes (46; 0.03%), recipient weight (14; 0.01%), and recipient height (8; 0.01%). Missing allograft-level values: cold ischemic time (842; 0.55%).

**Table 3.** Discrimination and Calibration for Machine Learning and Cox Model Analyses of the Primary Outcome of 3-Year Allograft Failure

	Model A (Limited to 8 Donor Variables in KDRI)^a^	Model B (KDRI + Additional Donor Variables)	Model C (Model B + Longitudinal Donor Biomarkers)	Model D (Model C + Recipient and Allograft Variables)	Model E (Model D + Donor/Recipient Interactions)	Model F (Model B + Recipient + Allograft Variables)	Model G (Model B + Recipient + Allograft Variables + Donor/Recipient Interactions)
Cox model							
iAUC	0.62	0.62	0.62	0.68	0.68	0.68^b^	0.67
Brier score	0.13	0.13	0.13	0.12	0.12	0.12^b^	0.12
LASSO							
iAUC	0.62	0.61	0.62	0.68	0.68	0.68	0.67
Brier score	0.13	0.13	0.13	0.12	0.12	0.12	0.12
Ridge							
iAUC	0.62	0.61	0.62	0.68	0.68	0.68	0.68
Brier score	0.13	0.13	0.13	0.12	0.12	0.12	0.12
Elastic net							
iAUC	0.62	0.61	0.62	0.68	0.68	0.68	0.67
Brier score	0.13	0.13	0.13	0.12	0.12	0.12	0.12
Random forest							
iAUC	0.57	0.57	0.58	0.63	0.63	0.65	0.65
Brier score	0.13	0.13	0.13	0.13	0.13	0.12	0.12

Abbreviations: iAUC, integrated (time-dependent) area under the curve; KDRI, Kidney Donor Risk Index; LASSO, least absolute shrinkage and selection operator.

a The Scientific Registry of Transplant Recipients report indicates that the new 8-variable KDRI has a C statistic (a widely used measure of discrimination) of approximately 0.59.^6,10^

b Final model: Kidney Allograft Survival Index.

**Table 4.** Variation in the Probability of 3-Year Graft Failure With the Kidney Allograft Survival Index Using Hypothetical Examples of 4 Kidney Donors and 8 Recipients

	Donor 1 (Small)		Donor 2 (Older)		Donor 3 (Young)		Donor 4 (Middle-Aged)	
	Recipient A	Recipient B	Recipient C	Recipient D	Recipient E	Recipient F	Recipient G	Recipient H
Recipient description	Small	Tall	Middle-aged	Older	Middle-aged	Older	Middle-aged, preemptive transplant	Older, dialysis
**Summary of donor and recipient profile**								
KDRI^a^	1.35	1.35	1.96	1.96	0.91	0.91	1.46	1.46
Predicted 3-y probability of graft failure with model A: 8 donor variables of KDRI	0.14	0.14	0.24	0.24	0.11	0.11	0.18	0.18
Predicted 3-y probability of graft failure using Kidney Allograft Survival Index	0.08	0.11	0.18	0.28	0.08	0.17	0.10	0.20
Predicted probability of delayed graft function (final DGF model)	0.07	0.11	0.22	0.25	0.11	0.14	0.26	0.42
**Donor characteristics**								
Donor age, y	40	40	58	58	35	35	45	45
Donor sex	F	F	F	F	M	M	F	F
Donor height, cm	152	152	152	152	178	178	178	178
Donor weight, kg	50	50	70	70	85	85	85	85
Donor hypertension	No	No	Yes	Yes	No	No	No	No
Donor history of diabetes	No	No	No	No	No	No	No	No
Donor cause of death	Drug overdose	Drug overdose	Stroke	Stroke	Drug overdose	Drug overdose	Stroke	Stroke
Donor smoking history	Yes	Yes	No	No	Yes	Yes	Yes	Yes
DCD	No	No	No	No	No	No	Yes	Yes
Donor terminal creatinine	1.0	1.0	1.8	1.8	1.0	1.0	1.5	1.5
**Allograft and recipient characteristics**								
Cold ischemia time, h	16	16	12	12	8	8	8	16
Transplant sidedness	Right	Right	Right	Right	Right	Right	Right	Right
Recipient age, y	50	50	45	70	40	68	40	65
Recipient sex	Female	Male	Female	Female	Male	Male	Male	Male
Dialysis duration d	1,095	1,095	1,095	1,095	1,095	1,095	0	1,825
Recipient height, cm	152	191	165	165	170	170	170	170
Recipient weight, kg	50	91	70	70	80	80	80	70
PRA	0	0	0	0	0	0	0	0
Prior transplant	No	No	No	Yes	No	No	No	No
Recipient diabetes	No	No	Yes	Yes	No	Yes	No	No
Transplant year	2020	2020	2020	2020	2020	2020	2020	2020
Recipient cause of ESRD	Cystic disease	Cystic disease	Diabetes	Diabetes	Hypertension	Diabetes	Hypertension	Hypertension

Abbreviations: DCD, donation after circulatory death; DGF, delayed graft function; ESRD, end-stage renal disease; KDRI, Kidney Donor Risk Index; PRA, panel-reactive antibodies.

a KDRI calculated using the new 8-variable equation.

## Data Availability

Data are available from the corresponding author upon reasonable request as allowed under IRB and federal guidelines.
