# Supplementary material for: Assessing Deceased-Donor Kidneys Through Posttransplant Survival Prediction Algorithms
Source: Am J Kidney Dis. Author manuscript; Available in PMC 2026 May 19. (PMC13185535; doi:10.1053/j.ajkd.2025.07.016)
Supplement: Supplementary Material — Figure S1: Magnified calibration plot for the Cox regression model for the primary outcome of 3-year all-cause graft failure (the Kidney Allograft Survival Index) and the primary cohort (20% testing split). Figure S2: Linear regression analysis of observed versus predicted 3-year all-cause graft failure (the Kidney Allograft Survival Index) and the primary cohort (20% testing split). Figure S3: Calibration plot for the Cox regression model for the outcome of 5-year all-cause graft failure in the primary cohort (20% testing split); a post hoc analysis. Item S1: Additional information about the dataset. Item S2: Imputation methods used to handle missing donor and recipient data. Item S3: Additional description of and rationale for machine-learning approaches. Item S4: Further description of performance metrics of discrimination and calibration. Table S1: List of donor and recipient variables tested. Table S2: Variables in the final models. Table S3: Cumulative incidence of outcomes among primary cohort of recipients with transplants recorded between April 15, 2016, and December 31, 2021, and secondary cohort of recipients with transplants recorded between May 1, 2007, and December 31, 2021. Table S4: Discrimination and calibration for machine-learning and logistic regression analyses of the delayed graft function outcome. Table S5: Discrimination and calibration for the secondary composite outcome of 1-year allograft failure and/or eGFR <20 mL/min/1.73 m2. [file NIHMS2172809-supplement-Supplementary_Material.pdf]

<u>Figure S1</u>: Magnified calibration plot for the Cox regression model for the primary outcome of three-year all-cause graft failure (the Kidney Allograft Survival Index) and the primary cohort (20% testing split)

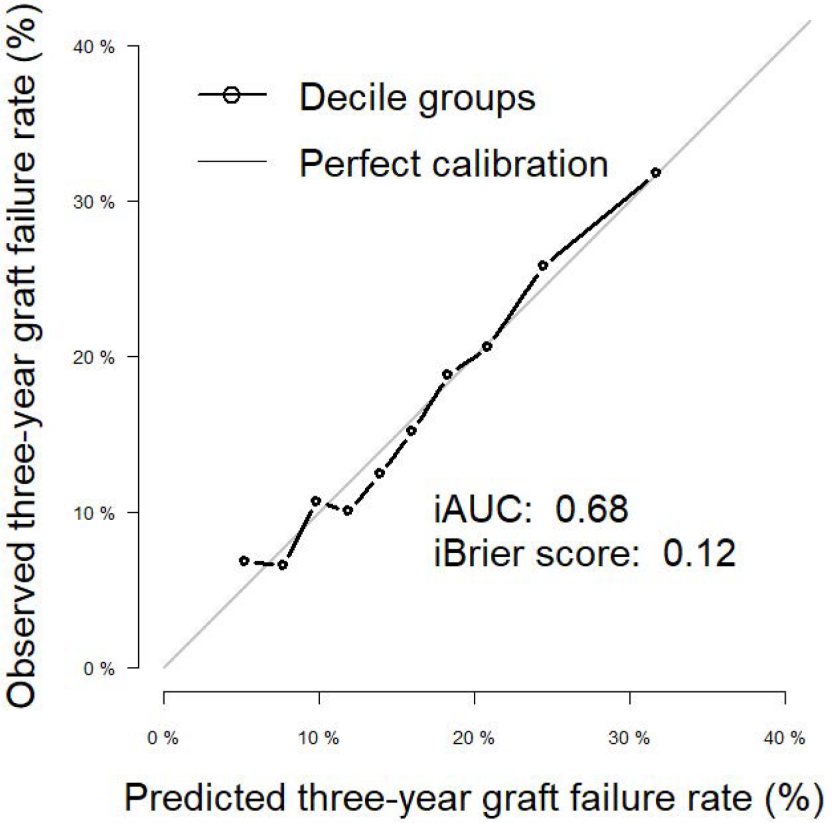

<u>Figure S2</u>: Linear regression analysis of observed vs. predicted three-year all-cause graft failure (the Kidney Allograft Survival Index) and the primary cohort (20% testing split).

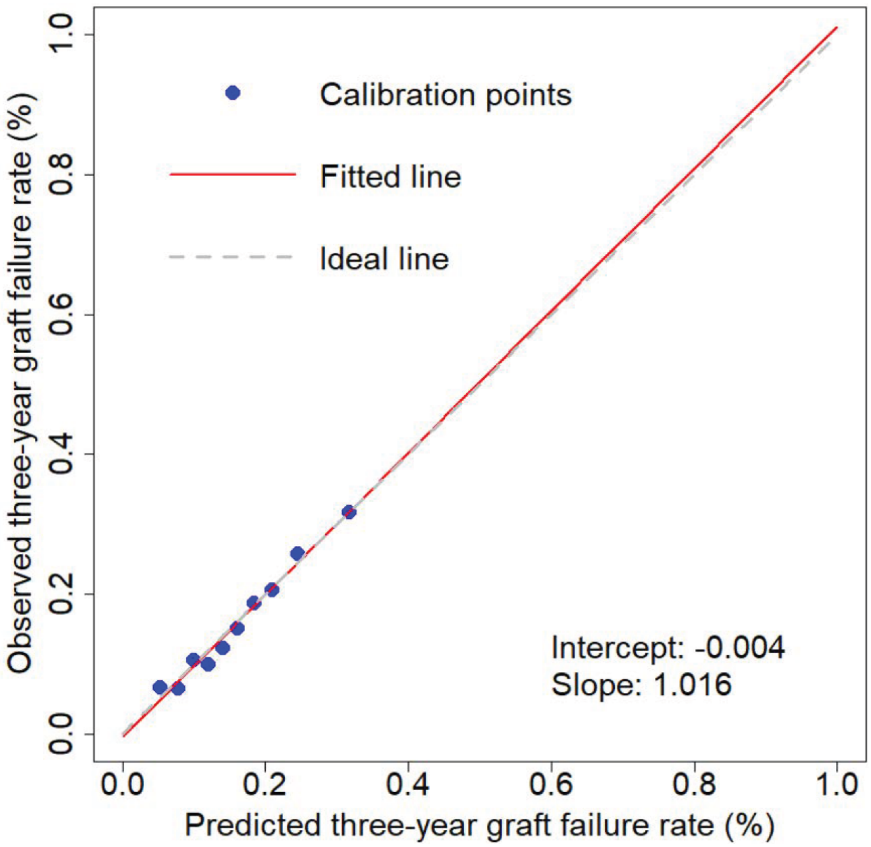

Figure S3: Calibration plot for the Cox regression model for the outcome of five-year all-cause graft failure in the primary cohort (20% testing split); a post-hoc analysis

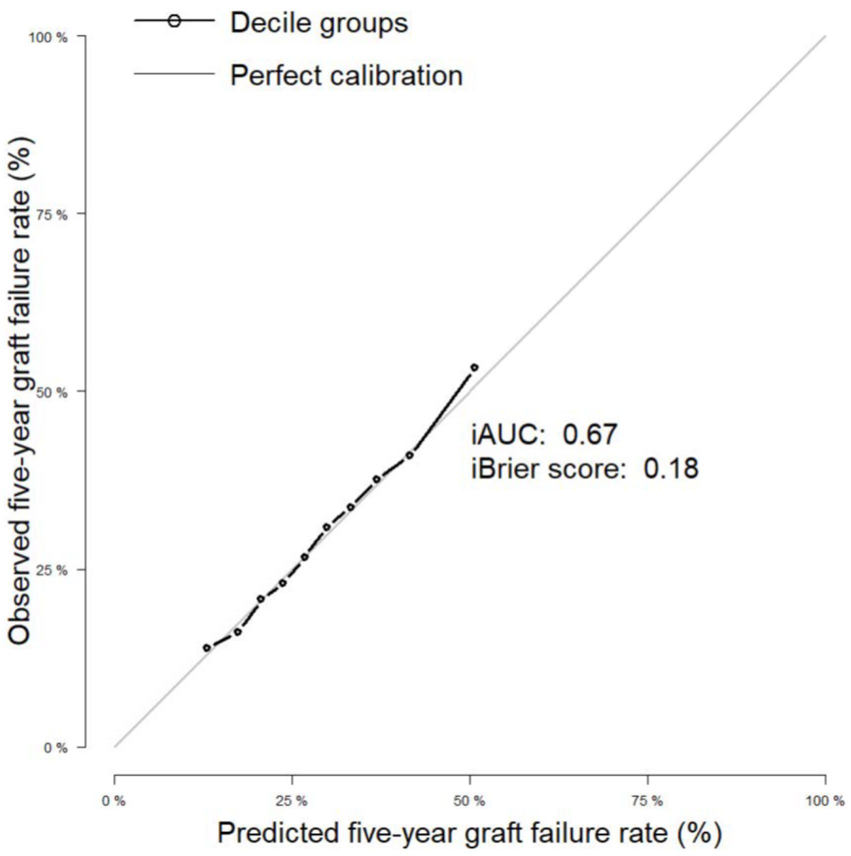

<u>Item S1</u>: Additional information about the dataset

We obtained a United Network for Organ Sharing (UNOS) Standard Analysis and Transplant Research (STAR) file and analyzed kidney transplants performed through December 31st, 2021. We subsequently received updated outcomes from UNOS on these recipients with outcomes reported through June 30, 2023. However, because outcomes reporting may lag at some centers, we censored follow-up at December 31, 2022.

UNOS disclaimer: The OPTN data system includes data on all donor, wait-listed candidates, and transplant recipients in the US, submitted by members of the Organ Procurement and Transplantation Network (OPTN). The Health Resources and Services Administration (HRSA), U.S. Department of Health and Human Services provides oversight to the activities of the OPTN contractor.

Item S2: Imputation methods used to handle missing donor and recipient data

The goal of this project was to create useful predictive tools for assessing allograft quality that could be made available to transplant clinicians for real-world use and that featured transparent methods. As a result, we elected not to address missing data with multiple imputation, an approach which may be more suitable for explanatory statistical models and which poses challenges when building data interfaces in which users can enter data for their own cases.

Instead, we opted to impute missing data as means or medians, for binary and continuous covariates, respectively; means and medians were computed within age quartiles, separately for donor- and recipient-specific covariates, since many variables and outcomes vary by age. Specifically, in the primary cohort, donor age quartiles were 29, 40, and 52 years while recipient age quartiles were 44, 55, and 64 years. Then, missing values were imputed accordingly. Note that for binary covariates, the mean value denotes the proportion of study participants with the condition in that specific age quartile; for inference tasks, this approach would not be easily interpretable, but for prediction tasks this is supported by every modeling approach considered in this study and provides an efficient, reproducible way of incorporating missing data within the algorithm, both at the training and validation/implementation stage.

Below, we provide an example of imputation values for height and weight for recipients in the primary cohort. For instance, a missing value for recipient height (HGT_CM_CALC) would be imputed to the value 168.9 for a recipient in the first age quartile (<44 years), and to 170.2 for a recipient in the second age quartile (44-55 years). The complete imputation data for all covariates with missing values is available upon request to the authors.

Covariate	Q1	Q2	Q3	Q4
HGT_CM_CALC	168.9	170.2	170.18	170
WGT_KG_CALC	77.5	85	82.5538	80.4

Item S3: Additional description of and rationale for machine learning approaches

For the Cox and logistic regression models, no tuning parameters needed to be selected. The node size for random forest was optimized over a grid of parameters from [1,$\sqrt{n}$] in increments of ln(n), where n is the total number of recipients in the cohort. For the random forest models, we used 1000 trees when predicting survival outcomes and 5000 trees when predicting binary outcomes. The rest of the parameters used in the random forest models were the default parameters for the randomForest function in the randomForest package in R when predicting binary outcomes or the rfsrc function in the randomForestSRC package in R when predicting survival outcomes.

Specifically, the default number of candidate variables sampled per split was $\sqrt{p}$, where p is the number of predictors and the maximum number of terminal nodes is the largest possible that can be achieved for the given node size. For the randomForest function, the minimum size of the terminal nodes was 1 for predicting binary outcomes, and for the rfsrc function the minimum size of the terminal nodes was 15 for predicting survival outcomes.

For the lasso, ridge, and elastic net methods, we used the default 100 regularization parameter values which are linear on the log scale and are generated automatically by the glmnet function in R for the given predictors and outcome which the algorithms were trained on. The largest regularization parameter for a given model that was considered was the one such that all coefficients are zero. The glmnet method will return fewer than 100 regularization parameter values if the model fit does not change sufficiently (measured by a fractional change in deviance of less than 10^{-5}) between consecutive lambdas or if the fraction of explained deviance reaches 0.999. All elastic net models used a mixing parameter of 0.5. The tuning parameters for lasso, ridge, elastic net and random forest selected for all outcomes for Model F are provided below for reference.

Machine learning method	Tuning parameter	Three-year all-cause graft failure	Delayed graft function	One-year allograft failure and/or eGFR <20
Lasso	Regularization parameter	3.9×10^{-4}	2.1×10^{-4}	2.6×10^{-4}
Ridge	Regularization parameter	4.06×10^{-3}	8.94×10^{-3}	2.7×10^{-3}
Elastic net	Regularization parameter	7.8×10^{-4}	3.9×10^{-4}	4.7×10^{-4}
Random forest	Node size	1	23	12

The lasso, ridge, elastic net, and random forest models were all trained on 80% of the full cohort and five-fold cross validation is used on the entire training cohort to select the regularization parameter based on minimum cross-validated error for lasso, ridge, elastic net, as well as the node size for the random forest. Our trained models with the optimal tuning parameters were then tested on the hold out 20% of the full cohort. Additionally, all interaction terms were removed before fitting the random forest models, as these models already account for interactions between predictors.

The lasso, ridge, and elastic methods were fit using cross-validation with AUC as the loss-function for the delayed graft function outcome and partial-likelihood for the allograft survival outcomes.

We chose lasso, ridge regression, and elastic net methods for our study because they are

among the simpler, more interpretable models which can handle data in high-dimensional settings. The ridge and elastic net models are well-suited to handle correlated predictors. Additionally, the lasso and elastic net methods shrink regression coefficients of non-informative predictors to zero. We also used random forest for our analyses to determine if it would provide improved predictive ability over the other methods due to its more flexible modelling of nonlinear interactions between predictors.

Item S4: Further description of performance metrics of discrimination and calibration

In evaluating the performance of our models, we evaluated our models' discrimination and calibration. For the binary outcome of delayed graft function (or the outcome of 1-year graft failure or 1-year eGFR ≤20 ml/min/1.73 m^2), the area under the receiver operating characteristic curve (AUC) was the measure of discrimination, which in essence, assessed how well our model predicted that allografts with delayed graft function had higher outcome probabilities than allografts without delayed graft function.[1,2] An AUC of 0.70, for example, would mean that our model correctly predicted which allograft was more likely to reach the outcome of delayed graft function 70% of the time. For the time-to-event outcome of all-cause graft failure by three years, we used the analogous integrated area under the time-dependent receiver operating characteristic curve (iAUC), which evaluates the model's predictive performance at each time point during follow-up and then summarizes across time.[1-3] Bootstrapping was used to estimate 95% confidence intervals: first, we resampled recipients with replacement until reaching the original sample size, then refit models and computed performance metrics in the bootstrap sample, repeated the process 1000 times, and then extracted the 0.025 and 0.975 quantiles of the resulting distribution.

In addition, we evaluated the equally important metric of calibration, which assesses, "how close a risk score's prediction of the expected outcome is to the observed outcome."[1] For example, if our model predicted that 30% of allografts with a given set of characteristics would survive for 3 years, but only 10% of those allografts actually survived 3 years, then it would suggest that our model is poorly calibrated, regardless of whether it correctly predicted that those allografts had higher probabilities of survival than allografts with a different set of characteristics (i.e., discrimination). We evaluated calibration graphically by comparing the predicted graft outcomes from our models to the observed outcomes (i.e., truth). Additionally, we calculated Brier scores, which is a quantitative summary measure of calibration to assess the agreement between predicted and observed outcomes, with lower Brier scores indicative of a better calibrated model.[1,4]

<u>Table S1</u>. List of donor and recipient variables tested

Donor characteristics from the kidney donor risk index:

Age, height, weight, hypertension, diabetes, cause of death: stroke, terminal creatinine, donation after circulatory death (DCD)

Additional donor variables:

Sex, smoking history

Donor-donor interactions:

Age/diabetes interaction, Sex/age interaction, age/creatinine interaction

Allograft characteristics:

Cold ischemia time, transplant sidedness

Recipient characteristics:

Age, sex, cause of renal disease, dialysis duration, height, weight, PRA, prior transplant, diabetes, transplant year

Donor, allograft, and recipient interactions:

CMV mismatch, donor/recipient age, donor/recipient height, donor/recipient sex, HLA mismatch, donor age and cold ischemia time

Donor longitudinal laboratory values from terminal hospitalization:

a. Creatinine (48 hr max/min/mean/SD/slope)
b. BUN (48 hr max/min/mean/SD/slope)
c. Bilirubin (48 hr max/min/mean/SD/slope)
d. Sodium (48 hr max/min/mean/SD/slope)
e. INR (48 hr max/min/mean/SD/slope)
f. SGOT (48 hr max/min/mean/SD/slope)
g. SGPT (48 hr max/min/mean/SD/slope)
h. Albumin (48 hr max/min/mean/SD/slope)

Variables not tested in final models, due to ethical concerns:

Recipient race, donor race, recipient socio-economic environment variables derived from the residence or insurance

Table S2: Variables in the final models

Model/Covariates	Model A	Model B	Model C	Model D	Model E	Model F	Model G
**Covariates**	**Model limited to 8 donor variables in the KDRI**	**KDRI + additional donor variables**	**Model B + longitudinal donor laboratory values**	**Model C + recipient and allograft variables**	**Model D + donor/recipient interactions**	**Model B + recipient + allograft variables**	**Model B + recipient + allograft variables + donor/recipient interactions**
**Donor age, donor height, donor weight, donor hypertension, donor diabetes, donor cause of death cerebrovascular/stroke, donor terminal creatinine, donor DCD status**	X	X	X	X	X	X	X
**Donor sex, donor history of smoking, donor age and diabetes interaction, donor age and donor sex interaction, donor age and donor terminal creatinine interaction**		X	X	X	X	X	X
**Longitudinal min/max/mean/standard deviation/slope (Albumin, BUN, creatinine, INR, SGOT, SGPT, Sodium, Bilirubin)**			X	X	X		
**Recipient age, recipient sex, recipient cause of end-stage renal disease, recipient dialysis duration, recipient height, recipient weight, PRA, recipient prior transplant, recipient diabetes status, transplant year**				X	X	X	X
**Cold ischemia time and transplant sidedness**				X	X	X	X
**CMV mismatch, donor and recipient age interaction, donor and recipient height interaction, donor and recipient sex interaction, HLA mismatch, donor age and cold ischemia time interaction**					X		X

Table S3: Cumulative incidence of outcomes among primary cohort of recipients with transplants recorded between April 15th, 2016, and December 31st, 2021, and secondary cohort of recipients with transplants recorded between May 1st, 2007, and December 31st, 2021

Outcome	Primary Cohort (N = 75,878)	Secondary Cohort (N = 157,884)
3-year all-cause graft failure*	9,509 (55.96); 11	21,677 (15.23); 17
1-year all-cause graft failure*	4,627 (63.99); 10	10,443 (19.04); 16
Delayed graft function**	22,751 (30.03%); 116	44,527 (28.22%); 122
1-year graft failure or eGFR ≤20 ml/min/1.73 m^2 ***	5,790 (7.63%); 10	12,895 (8.17%); 16

* Values are counts with event rate per 1,000 person-years (for 3-year and 1-year all-cause graft failure) and counts with proportions (for the other two outcomes), with number of missing outcome values after the semi-colon.
** Delayed graft function is defined as dialysis within the first 7 days after transplantation.
*** 1-year graft failure or eGFR < ≤20 ml/min/1.73 m^2 is a binary outcome assessed at 1 year.

Table S4: Discrimination and calibration for machine learning and logistic regression analyses of the delayed graft function (DGF) outcome

Analytic Method	Performance Metric	Model A	Model B	Model C	Model D	Model E	Model F	Model G
		Model limited to 8 donor variables in the KDRI*	KDRI + additional donor variables	Model B + longitudinal donor laboratory values	Model C + recipient and allograft variables	Model D + donor/recipient interactions	Model B + recipient + allograft variables	Model B + recipient + allograft variables + donor/recipient interactions
Logistic regression	AUC	0.70	0.70	0.70	0.75	0.75	0.75*	0.75
	Brier Score	0.19	0.19	0.19	0.18	0.18	0.18*	0.18
Lasso	AUC	0.70	0.70	0.70	0.75	0.75	0.75	0.75
	Brier Score	0.19	0.19	0.19	0.18	0.17	0.18	0.18
Ridge	AUC	0.70	0.70	0.71	0.75	0.75	0.74	0.75
	Brier Score	0.19	0.19	0.19	0.18	0.18	0.18	0.18
Elastic net	AUC	0.70	0.70	0.70	0.75	0.75	0.75	0.75
	Brier Score	0.19	0.19	0.19	0.18	0.17	0.18	0.18
Random Forest	AUC	0.69	0.69	0.71	0.76	0.76	0.76	0.76
	Brier Score	0.38	0.35	0.34	0.31	0.31	0.34	0.34

*Final DGF Model

Table S5: Discrimination and calibration for the secondary composite outcome of 1-year allograft failure and/or eGFR <20 ml/min1.73 m^2

Analytic Method	Performance Metric	Model A	Model B	Model C	Model D	Model E	Model F	Model G
		Model limited to 8 donor variables in the KDRI*	KDRI + additional donor variables	Model B + longitudinal donor laboratory values	Model C + recipient and allograft variables	Model D + donor/recipient interactions	Model B + recipient + allograft variables	Model B + recipient + allograft variables + donor/recipient interactions
Logistic regression	AUC	0.64	0.64	0.64	0.68	0.68	0.68	0.68
	Brier Score	0.07	0.07	0.07	0.07	0.07	0.07	0.07
Lasso	AUC	0.64	0.64	0.64	0.68	0.68	0.68	0.68
	Brier Score	0.07	0.07	0.07	0.07	0.07	0.07	0.07
Ridge	AUC	0.64	0.64	0.64	0.68	0.68	0.68	0.68
	Brier Score	0.07	0.07	0.07	0.07	0.07	0.07	0.07
Elastic net	AUC	0.64	0.64	0.64	0.68	0.68	0.68	0.68
	Brier Score	0.07	0.07	0.07	0.07	0.07	0.07	0.07
Random Forest	AUC	0.60	0.61	0.61	0.65	0.65	0.67	0.67
	Brier Score	0.49	0.47	0.45	0.45	0.45	0.45	0.45

References

1. Goldberg D, Ishwaran H, Potluri V, et al. Evaluating allograft risk models in organ transplantation: Understanding and balancing model discrimination and calibration. *Liver Transpl*. Jan 31 2025;doi:10.1097/LVT.0000000000000575
2. Hartman N, Kim S, He K, Kalbfleisch JD. Pitfalls of the concordance index for survival outcomes. *Stat Med*. Jun 15 2023;42(13):2179-2190. doi:10.1002/sim.9717
3. Heagerty PJ, Zheng Y. Survival model predictive accuracy and ROC curves. *Biometrics*. Mar 2005;61(1):92-105. doi:10.1111/j.0006-341X.2005.030814.x
4. Steyerberg EW, Vickers AJ, Cook NR, et al. Assessing the performance of prediction models: a framework for traditional and novel measures. *Epidemiology*. 2010;21(1):128-138.
5. Organ Procurement and Transplantation Network. Public Comment Proposal. Refit Kidney Donor Profile Index without Race and Hepatitis C Virus. OPTN Minority Affairs Committee. Accessed 10/31/2024. URL: https://optn.transplant.hrsa.gov/media/ekkfxy4t/mac_kdpiracehcv_pcjan24.pdf
